# Supplementary material for: The interactome of intact mitochondria by cross-linking mass spectrometry provides evidence for coexisting respiratory supercomplexes
Source: Mol Cell Proteomics. 2017 Dec 8;17(2):216–32. doi: 10.1074/mcp.RA117.000470 (PMC5795388; doi:10.1074/mcp.RA117.000470)
Supplement: Supplemental Data [file supp_RA117.000470_133922_0_supp_23978_fzffwf.zip › spectra_annotation/mito_spectra_annotation/64-1-34-1-3-1.pdf]

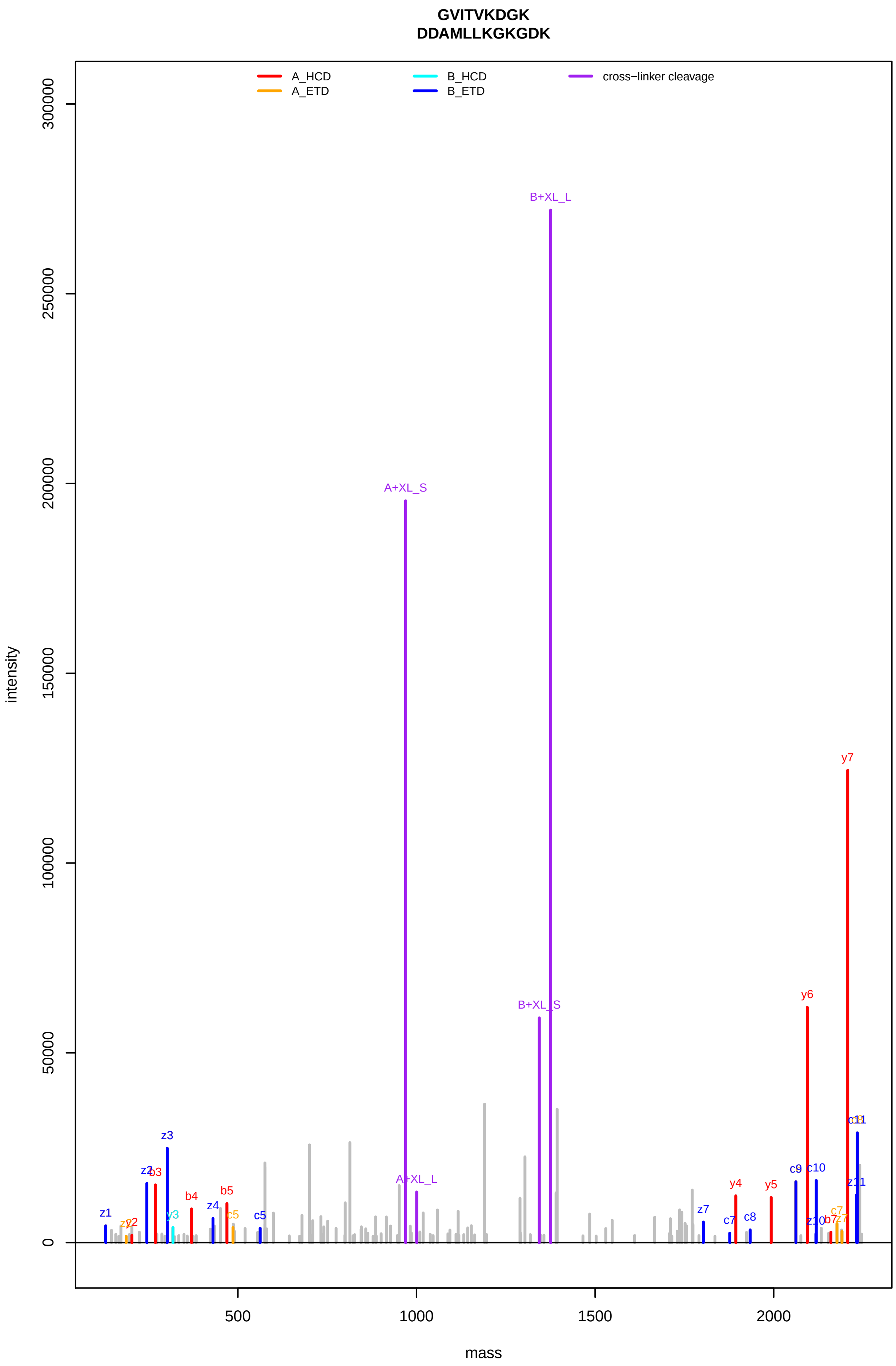
GVITVKDGK
DDAMLLKGKGDK
A_HCD
A_ETD
B_HCD
B_ETD
cross−linker cleavage
intensity
mass
B+XL_L
A+XL_S
B+XL_S
A+XL_L
y7
y6
y5
y4
c11
c10
c9
z11
z10
z7
c8
c7
c5
b5
b4
b3
z3
z4
z2
z1
y3
y2
b7
c7

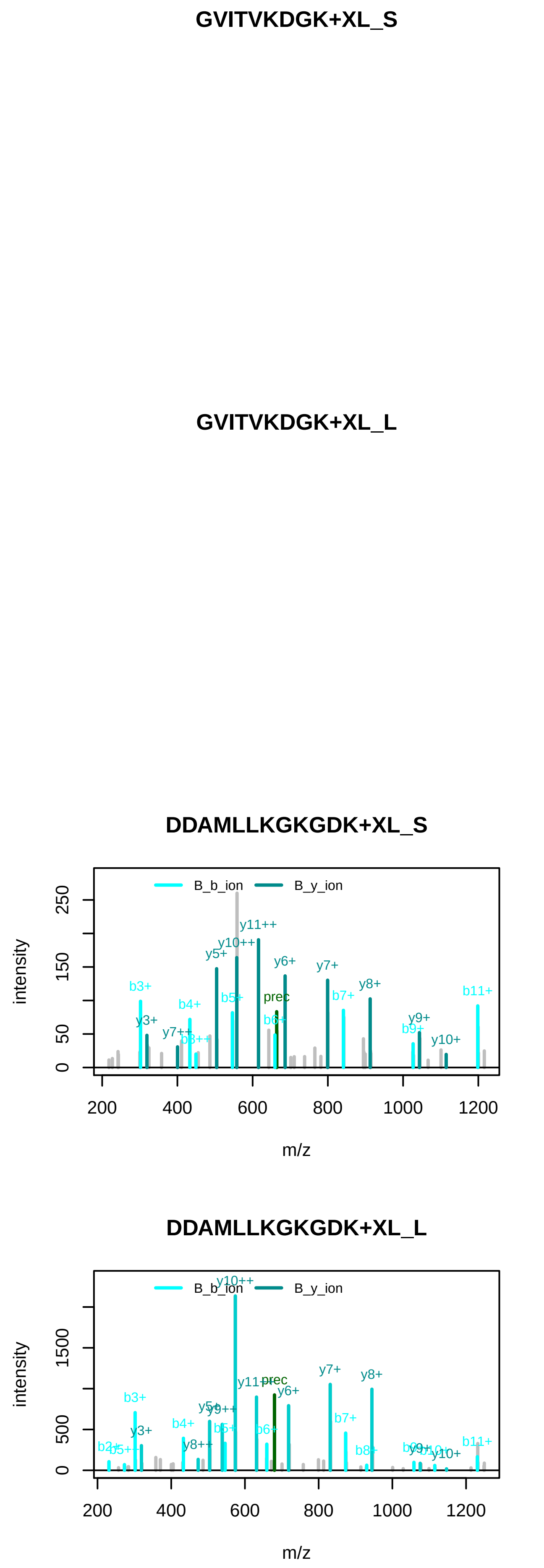
GVITVKDGK+XL_S
GVITVKDGK+XL_L
DDAMLLKGKGDK+XL_S
B_b_ion
B_y_ion
intensity
m/z
b3+
y3+
b4+
y5+
y10++
y11++
b5+
y6+
prec
y7+
b7+
y8+
y9+
b9+
y10+
b11+
DDAMLLKGKGDK+XL_L
y10++
y11++
prec
y6+
y7+
y8+
b7+
b3+
b4+
y3+
b11+
